# Erythropoietin regulates immune/inflammatory reaction and improves neurological function outcomes in traumatic brain injury

**DOI:** 10.1002/brb3.827

**Published:** 2017-10-03

**Authors:** Zi‐wei Zhou, Fei Li, Zhi‐tong Zheng, Ya‐dan Li, Tong‐heng Chen, Wei‐wei Gao, Jie‐li Chen, Jian‐ning Zhang

**Affiliations:** ^1^ Department of Neurosurgery Tianjin Medical University General Hospital Heping District Tianjin China; ^2^ Tianjin Neurological Institute Tianjin China; ^3^ Key Laboratory of Post‐trauma Neuro‐repair and Regeneration in Central Nervous System Ministry of Education Heping District Tianjin China; ^4^ Tianjin Key Laboratory of Injuries, Variations and Regeneration of Nervous System Heping District Tianjin China; ^5^ Intensive Care Units Tianjin Huanhu Hospital Tianjin China; ^6^ Department of Neurosurgery The Second Hospital Tianjin Medical University Hexi District Tianjin China; ^7^ Department of Neurology Henry Ford Hospital Detroit MI USA

**Keywords:** erythropoietin, neuroimmune, neuroinflammation, regulatory T cell (Treg), traumatic brain injury

## Abstract

**Introduction:**

Traumatic brain injury (TBI) remains a leading cause of disability and death among young people in China. Unfortunately, no specific pharmacological agents to block the progression of secondary brain injury have been approved for clinical treatment. Recently, neuroprotective effects of erythropoietin (EPO) have been demonstrated in addition to its principal function in erythropoiesis, and hence it is viewed as a potential drug for TBI. In this study, we have investigated the neuroprotective effects of EPO associated with immune/inflammatory modulation in a mouse experimental TBI model.

**Methods:**

EPO (5000 U/kg body weight, i.p.) was injected at 1 hr, 1, 2, and 3 days after TBI, and its effect on cognitive function, brain edema, immune/inflammatory cells including regulatory T cells (Tregs), neutrophils, CD3^+^ T cells, and microglia, cytokines including interleukin‐10 (IL‐10), transforming growth factor‐β (TGF‐β), interleukin‐1β (IL‐1β), and tumor necrosis factor‐α (TNF‐α) were evaluated at different time points after treatment.

**Results:**

EPO treatment significantly decreased brain edema and improved cognitive function when compared to Saline‐treated mice (*p *<* *.05). EPO treatment also significantly increased Tregs level in spleen and injured brain tissue as well as significantly reduced the infiltration and activation of immune/inflammatory cells (neutrophils, CD3^+^T cells, and microglia) in the injured hemisphere compared to Saline‐treated control animals (*p *<* *.05). In addition, ELISA analysis demonstrated that EPO treatment increased the expression of anti‐inflammatory cytokine IL‐10, but decreased the expression of proinflammatory cytokine IL‐1β and TNF‐α in the injured brain tissue (*p *<* *.05).

**Conclusions:**

These findings suggest that EPO could improve neurological and cognitive functional outcomes as well as regulate immune/inflammatory reaction in TBI.

## 1 INTRODUCTION

Traumatic brain injury (TBI) is a leading cause of mortality, morbidity, and in China it is the highest contributor to mortality in adults under 40 years of age (Wu et al., 2008). In addition to primary damages to the brain that occurs immediately following injury, secondary brain injury is highly heterogeneous in presentation and multifactorial in causes (Bramlett & Dietrich, 2004). A key feature of secondary brain injury is cerebral inflammation, which is characterized by microglial activation, upregulation and secretion of chemokines and cytokines, infiltration of peripheral immune cells including neutrophils, T lymphocytes, B lymphocytes, and NK cells (Roberts et al., 2013). Despite earnest research, no effective therapy has been developed for the treatment of TBI at present. Till date, a multitude of neuroprotective agents have been investigated for TBI treatment in the laboratory, including tumor necrosis factor‐α (TNF‐α) antagonist (Baratz et al., 2011), free radical scavengers (Aiguo, Zhe, & Gomez‐Pinilla, 2010), the nicotinic acetylcholine‐receptor activator donepezil and mexiletine (Atalay, Caner, Can, & Cekinmez, 2007; Fujiki et al., 2008), but none have been successful in clinical studies. To our knowledge, one of the reasons for this may be that these agents target a specific and restricted pathophysiological injury mechanism, while the injury mechanism of TBI is complex.

Erythropoietin (EPO), a glycoprotein of 165 amino acids, belongs to the cytokine superfamily and has been viewed as the hematopoiesis‐regulating hormone. Under the control of oxygen‐sensing hypoxia inducible factor (HIF) pathway (Yi & Hak, 2012), EPO is predominantly produced by the interstitial fibroblasts of the renal cortex and outer medulla in adult animals, and by Kuffer's cells in the liver during embryonic development (Tan, Eckardt, & Ratcliffe, 1991). It has been widely used to treat chronic renal‐failure patients with anemia for many years in clinic (Teixeira et al., 2010). Recently, apart from its principal function in erythropoiesis, nonhematopoietic functions of EPO such as angiogenic, cardioprotective, and neuroprotective effects have attracted extensive concerned (Kertesz, Wu, Chen, Sucov, & Wu, 2004; Kumral et al., 2011; Tada et al., 2006). Erythropoietin receptor (EPOR) is expressed by a variety of nonhematopoietic cell types such as vascular smooth muscle cells, myocardial cells, brain capillary endothelial cells, and neurons (Acheson, Richards, & de Wit, 2007; Bernaudin et al., 1999; Cianferotti & Brandi, 2014; Wright et al., 2004). EPO can alleviate motor and cognitive deficit, axonal pathology, and neuroinflammation in the models of cerebral ischemia (Villa et al., 2003), experimental autoimmune encephalomyelitis (EAE) (Yuan et al., 2008), and diffuse axonal injury (DAI) (Hellewell, Yan, Alwis, Bye, & Morganti‐Kossmann, 2013). Immune/inflammatory responses play an important role in the pathological process of secondary brain injury. However, there are only a few studies investigating the role of EPO in the regulation of immune/inflammatory responses following TBI.

In this study, we demonstrate that EPO treatment can improve neurological functional outcomes in a well‐standardized experimental model of TBI in mice, and hypothesize that its protective roles are associated with immune/inflammatory modulation. The therapeutic effects of EPO in TBI model and the changes in regulatory T cells (Tregs), neutrophils, CD3^+^ T cell, microglia, and some cytokines are investigated in this study.

## 2 MATERIAL AND METHODS

### 2.1 Subjects and fluid percussion injury (FPI) model

Adult male C57BL/6 mice (23–25 g) (HFK Bioscience, Beijing, China) were maintained in 12 hr light–dark cycles and ad lib access to food and water. All mice were rigorously treated in accordance to the guidelines of the Chinese Small Animal Protection Association and this research protocol was approved by the Ethics Committee of Tianjin Medical University General Hospital. Adult male C57BL/6 mice were weighed and anesthetized with 10% chloride hydrate (3.0 ml/kg) administered via i.p. injection. The core temperature of experimental mice was maintained at 37°C by a heating pad during surgery. Mice were fixed in a stereotaxic frame. The hair on the head of mice was removed and sterilized with ethanol. The skull was exposed by midline scalp incision so that craniotomy (3 mm diameter) was performed over right parietal bone. Surgical sites were selected by 2.0 mm right of the sagittal suture and 2.0 mm off of the coronal suture using a dental drill. The dura mater was kept intact during surgery. A female Luer‐lok, filled with saline, was rigorously fixed on the surgical site. A tube of the LFP device (New Sun Health Products, Cedar Bluff, VA, USA) with a male Luer‐lok was tightly connected to the female Luer‐lok fitting of the hub. When anesthesia dissipated, mice were subjected to experimental FPI at a pressure of 2.0 atmosphere (atm). Mice were sterilized and sutured after their vital signs were stable and then returned to the home cage (Carbonell, Maris, McCall, & Grady, 1998).

### 2.2 Erythropoietin treatment protocol and experimental groups

Male C57BL/6 mice (23–25 g) were randomly divided into three groups. (1) Sham group: mice underwent surgery without FPI; (2) TBI + Saline control group: mice received FPI and were treated with Saline; and (3) TBI + EPO treatment group: mice received FPI and were treated with EPO. Recombinant human EPO was provided by Roche Diagnostics GmbH (Mannheim, Germany). At 1 hr and 1, 2, and 3 days after FPI, mice were injected intraperitoneally with EPO (5000 U/kg) or equal volume of sterile saline (Chen et al., 2007; Villa et al., 2003).

### 2.3 Brain edema assessment

Mice were anesthetized and sacrificed on 1, 3, and 7 days after TBI (*n *= 6/group) to obtain the brain, which was then divided along the midline. The ipsilateral brain was weighed immediately to obtain wet weight. They are then dried at 65°C for 72 hr to measure the dry weight. Water content in the brain was calculated as: water content (%) = (wet weight‐dry weight)/wet weight × 100 (Xu, Yu, Ding, & Zheng, 2012).

### 2.4 Flow cytometry

Mice were sacrificed at 1, 3, and 7 days after TBI (*n *= 6/group). The spleen was surgically removed. After washing to remove blood, the spleen was grinded and homogenized. The tissue suspension was centrifuged at 300 g for 15 min at 4°C. The supernatant is removed and the cell pellet is resuspended in the Fetal Bovine Serum buffer to obtain mononuclear cell suspension. Tregs are labeled using a commercial Tregs Isolation kit (eBioscience, San Diego, CA, USA) including antimouse CD4 and Foxp3 antibodies and analyzed by FACSAria™ flow cytometer (BD, Biosciences, USA). Diva™ analysis program was used to quantify experimental data.

### 2.5 Immunohistochemistry

At 1, 3, and 7 days after injury (early stages of TBI), mice were anesthetized with chloride hydrate (3.0 ml/kg) and perfused transcardially with 0.9% saline followed by 4% of paraformaldehyde (in 0.1 mol/L PBS, pH7.4). The brain was removed immediately after perfusion and fixed in 4% paraformaldehyde for 24 hr at room temperature. The fixed brain was embedded in paraffin and 6 μm sections were made from these paraffin tissue blocks (*n *= 3/group).

For immunohistochemistry staining, brain paraffin sections were deparaffinized and rehydrated. Heat antigen retrieval was performed in a citrate buffer (pH 6.0) for 20 min. These sections were washed with PBS 3 × 5 min and incubated with 3% BSA to block unspecific binding and penetrated membrane with 0.3% Triton‐X‐100. Then, they were incubated with primary rabbit anti‐Foxp3, anti‐CD3, or antimyeloperoxidase (MPO) antibodies (Abcam, Cambridge, UK) overnight at 4°C. The binding of these primary antibodies was recognized by a goat‐anti‐rabbit IgG (ZSGB‐BIO Co., Ltd. Beijing, China) 60 min at 37°C. Diaminobenzidine (ZSGB‐BIO Co., Ltd.) was used as a sensitive chromogen. Slides were counterstained with hematoxylin.

In addition, frozen sections were also prepared and incubated with anti‐IBA1 (microglia marker) antibody (Abcam) overnight at 4°C. The sections were washed three times with PBS for 5 min each and then incubated with PE‐IgG (1:500, Cell Signaling Technology) at 37°C for 1 hr. After washing another three times, the sections were counterstained with DAPI (1:5000; Sigma‐Aldrich, St. Louis, MO, USA) for 10 min to stain the nucleus, washed again, and mounted with glycerol (*n *= 3/group).

The numbers of Foxp3‐positive, MPO‐positive, CD3‐positive, and IBA‐positive cells in each section were counted (per 100×, Nikon, Japan) in five fields in the injured hemisphere by two independent observers who were blind to the study conditions, to obtain an average number of the counted cells per view field.

### 2.6 Enzyme‐linked immunosorbent assay (ELISA)

To quantify the release of cytokines from the brain after TBI (*n *= 6/group), mice were sacrificed at 1, 3, and 7 days after TBI, brain homogenates were centrifuged at 300 g for 15 min at 4°C. The cytokines IL‐10, TGF‐β, IL‐1β, and TNF‐α were quantified in the supernatant used commercial ELISA kits (eBioscience). All procedures are performed according to manufacturers’ protocols.

### 2.7 Morris Water Maze

To test the cognitive functional outcome, Morris Water Maze (MWM) was performed in another set of animals as previously described (Vorhees & Williams, 2006) from 7 to 11 days after TBI (*n *= 10/group). Briefly, the maze (120 cm diameter, 40 cm high; DMS‐2, Chinese Academy of Sciences, China) was filled with water (19–22°C) and nontoxic white paint. A platform (8 cm diameter) was located 0.5 cm below water surface in the center of southwest quadrant. Spatial learning was tested by recording the time for finding the hidden platform at 1 week postinjury. Each mouse was trained with four daily trials for 5 consecutive days. Then, a probe trail was executed to assess the memory retention when the platform was removed on day 6 (12 days after TBI). We recorded the time the mice stayed in the goal quadrant during a 30s period.

### 2.8 Statistical analysis

Data are expressed as mean ± SD and analyzed by SPSS16.0 software. The data of spatial acquisition in MWM tests were analyzed by repeated measures ANOVA and one‐way ANOVA. The other data were analyzed by one‐way ANOVA with least significance difference (LSD). Statistical significance is defined as *p *<* *.05.

## 3 RESULTS

### 3.1 EPO‐reduced brain edema induced by TBI

**Figure 1:**
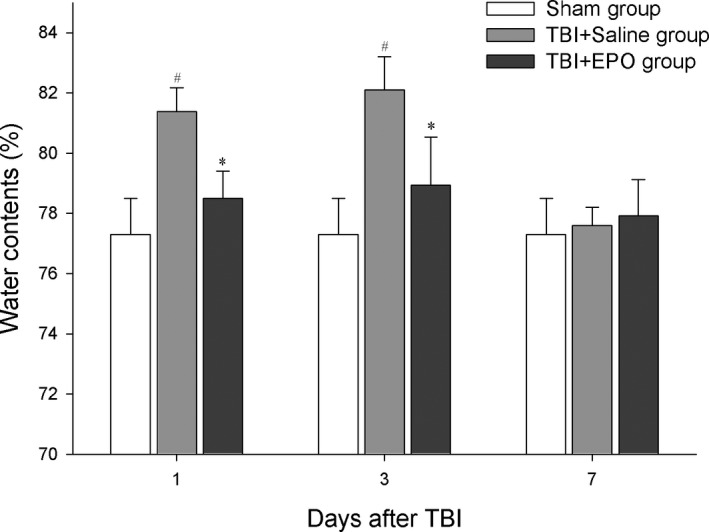
Brain edema measured through water content measurement. TBI significantly increased brain edema at 1 and 3 days after TBI; EPO treatment significantly decreased brain edema at 1 and 3 days after TBI compared to TBI + Saline control. *n *= 6/group, *p < .05 TBI + EPO group versus TBI + Saline group, #*p* < .05 versus Sham group

The changes in water content in the ipsilateral hemisphere, which could represent conditions of brain edema, were detected at 1, 3, and 7 days after TBI. TBI + Saline mice showed increased brain water content on 1 and 3 days after TBI compared to sham control group (*p *<* *.05). As compared to the saline‐treated group, EPO‐treated mice showed significantly decreased brain water content on 1 and 3 days post‐TBI (*p *<* *.05, Figure 1).

### 3.2 EPO treatment of TBI increased peripheral and brain Tregs levels

**Figure 2:**
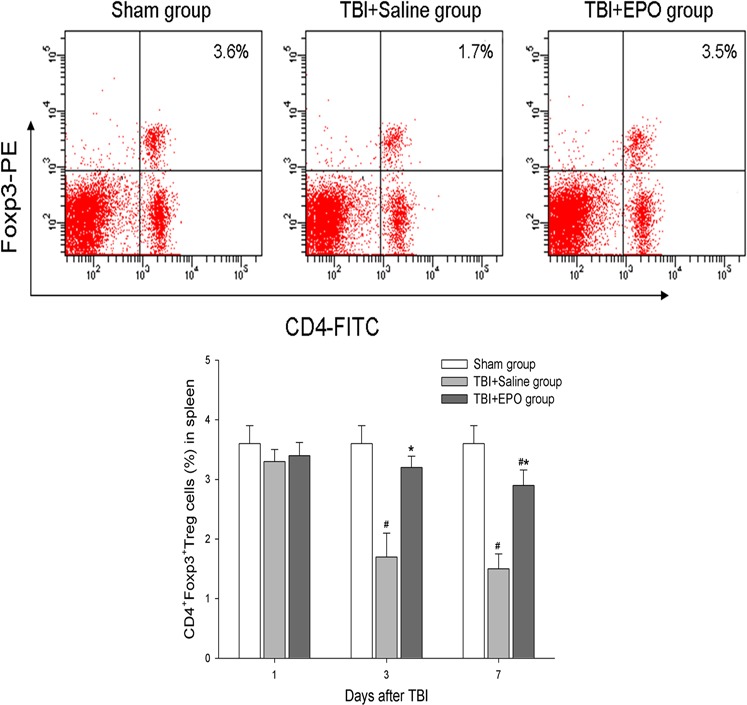
Detect the changes in Treg in spleen by FACS. Tregs were stained by CD4 and Foxp3 antibodies. After mononuclear cells being gated, the double positive cells in outer and upper quadrant were defined as Tregs. TBI significantly decreased the level of Tregs at 3 and 7 days after injury, but EPO treatment significantly increased Tregs level at 3 and 7 days after injury. *n *= 6/group, *p < .05 TBI + EPO group versus TBI + Saline group, #*p* < .05 versus Sham group

To test whether EPO regulates peripheral Tregs after TBI, CD4 and Foxp3 antibody were employed to measure spleen Treg by flow cytometry at 1, 3, and 7 days. The double positive cells in outer and upper quadrant represent Treg cells (Figure 2). The changes in Treg cells have no difference in each group at 1 day after TBI. At 3 and 7 days after TBI, the percents of Treg cells in mononuclear cells of spleen significantly decreased in the TBI control compared with Sham control group (*p *<* *.05). Moreover, the levels of Treg in TBI + EPO treatment group are significantly increased compared to TBI + Saline group (*p *<* *.05).

**Figure 3:**
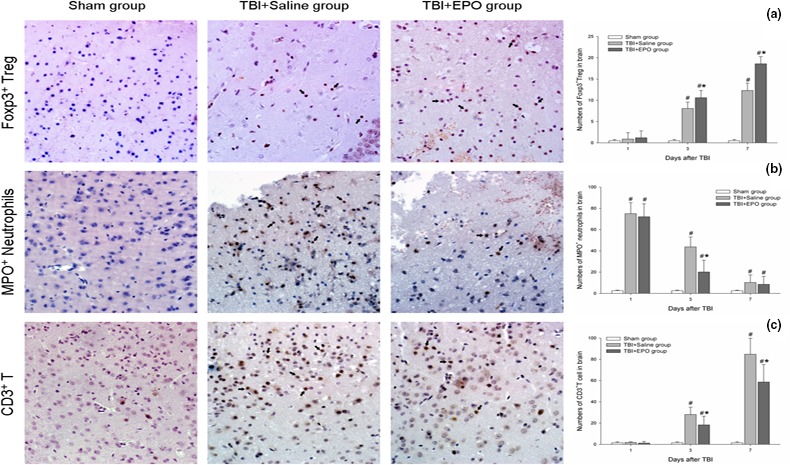
Detect the changes in immuno/inflammatory cells in injured brain tissue by immunohistochemistry staining. The positive cells are brown and directed by black arrowheads. (a) Foxp3+ Tregs mainly appeared around the lesions at 3 and 7 days after TBI. EPO treatment increased Foxp3+ Tregs expression in the injured brain. (b) MPO+ neutrophils promptly appeared in and around lesions at 1 day after TBI, and then gradually decreased. Compared with TBI + Saline group, EPO treatment significantly decreased numbers of MPO +  neutrophils at 1 and 3 days after TBI. (c) TBI increased the infiltration of CD3+ T cells in the injured brain tissue at 3 and 7 days after TBI. EPO treatment significantly decreased CD3+ T cells levels compared with TBI + Saline group. *n *= 3/group, *p < .05 TBI + EPO group versus TBI + Saline group, #*p* < .05 TBI + EPO group or TBI + Saline group versus Sham group

To test whether EPO regulates brain tissue Tregs after TBI, Foxp3^+^ Treg cell immunohistochemical staining was performed in the brain section (Figure 3a). Our data show that the Foxp3^+^ Treg cells mainly appear at 3 and 7 days in TBI + EPO and TBI + Saline group. But there were no Foxp3^+^ Treg cells found in Sham group at any time point. In addition, EPO treatment TBI mice exhibits significantly increased Foxp3^+^ Treg cells around the injured brain tissue compared to the TBI + Saline group at 3 and 7 days after TBI (*p *<* *.05). The data indicate that EPO treatment significantly increases Treg expression in spleen and in the injured brain tissue.

### 3.3 EPO attenuated the infiltration and activation of immune/inflammatory cells

In order to observe the infiltration of peripheral immune/inflammatory cells after TBI, myeloperoxidase (MPO, neutrophils marker) and CD3 (CD3 T cell) antibody were employed to identify neutrophils or CD3^+^ T cells at 1, 3, and 7 days. Here, we show that the MPO^+^ neutrophils primarily appeared in and around lesions of injured hemisphere at 1 and 3 days after TBI (Figure 3b). While CD3^+^ T‐cell distribution is different from neutrophils, CD3^+^ T cells mainly appeared around the lesions at 3 and 7 days (Figure 3c). EPO treatment of TBI significantly decreased the numbers of MPO^+^ neutrophils as well as CD3^+^ T cells in the injured brain compared with TBI + Saline group (*p *<* *.05).

**Figure 4:**
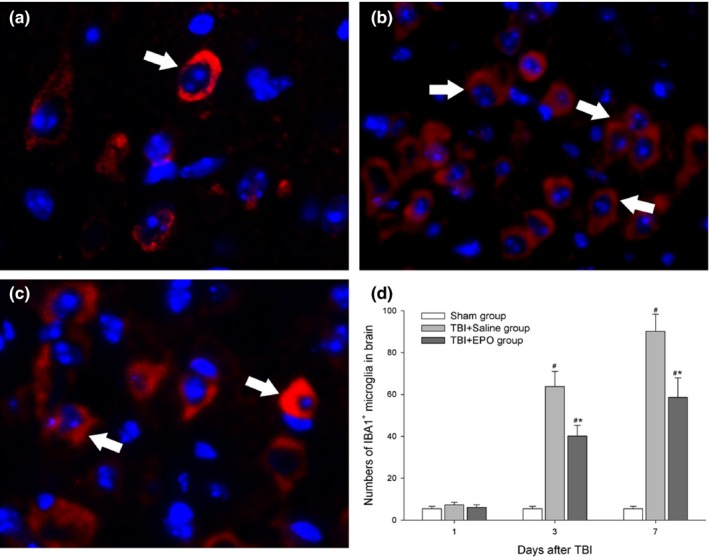
Detect the activation of microglia in injured hemisphere by immunofluorescence staining. The microglia was stained by IBA1 antibody (red) and cell nucleus was stained by DAPI (blue). The cells directed by white arrowheads are positive cells. (a) Sham group, (b) TBI + Saline group, (c) TBI + EPO group, (d) TBI increased IBA1 expression in the injured brain compared to sham control; EPO treatment significantly decreased IBA1 expression at 3 and 7 days after TBI. *n *= 3/group, *p < .05 TBI + EPO group versus TBI + Saline group, #*p* < .05 TBI + EPO group or TBI + Saline group versus Sham group

We use IBA1 (active microglia cell marker) antibody to identify the activation of inherent immune‐related cells in brain. Figure 4 shows that there are more numbers of activated microglia in TBI + EPO and TBI + Saline group than that in Sham group. IBA1‐positive cells mainly appeared around the lesions at 3 and 7 days after TBI. Moreover, as compared to TBI + Saline group, TBI + EPO treatment group has significantly less numbers of activated microglia in the ipsilateral brain tissue (*p *<* *.05, Figure 4).

### 3.4 EPO reduced proinflammatory and increased anti‐inflammatory cytokines

**Figure 5:**
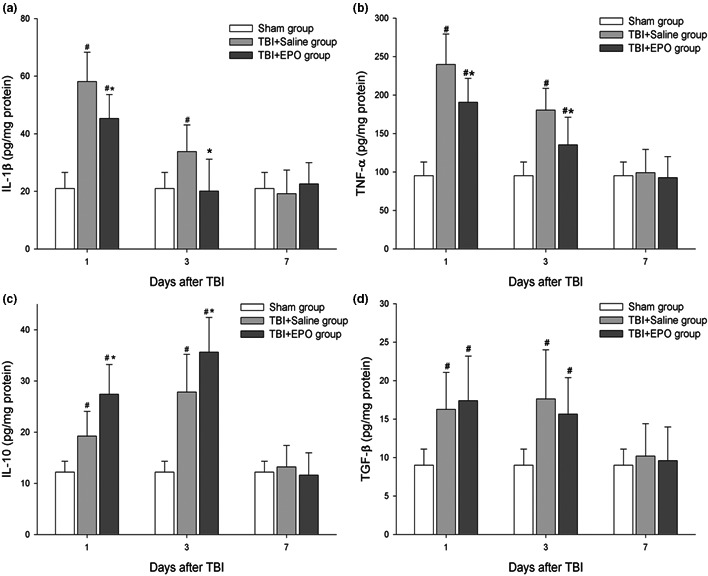
Detect immuno/inflammatory cytokines in the brain tissue of injured hemisphere by ELISA at 1, 3, and 7 days after TBI. (a and b), respectively, show that TBI increased the expression of proinflammatory cytokines IL‐1β and TNF‐α at postinjury 1 and 3 days compared to sham control group; EPO treatment of TBI significantly decreased IL‐1β and TNF‐α levels at 1 and 3 days after TBI compared to TBI control group. (c) EPO treatment increased the expression of anti‐inflammatory cytokine IL‐10 after TBI compared to TBI + Saline group. (d) There was no significant difference between EPO treatment and TBI + Saline on the regulation of TGF‐β expression. *n *= 6/group, *p < .05 TBI + EPO group vs. TBI + Saline group, #*p* < .05. TBI + EPO group or TBI + Saline group versus Sham group

To test whether EPO treatment regulates proinflammatory and anti‐inflammatory factor levels in the injured brain tissue, the changes in IL‐1β, TNF‐α, IL‐10, and TGF‐β in brain tissue were measured. Brain homogenates from the injured hemisphere were collected and measured by ELISA at 1, 3, and 7 days (Figure 5). Our data show that the trauma stress induced by TBI upregulates expression of the above‐mentioned cytokines at 1 and 3 days after TBI. EPO treatment of TBI significantly suppressed the expression of proinflammatory cytokines including IL‐1β and TNF‐α at 1 and 3 days compared to TBI + Saline control group (*p *<* *.05). Also, EPO treatment of TBI significantly increased the expression of anti‐inflammatory cytokines IL‐10, but not TGF‐β at 1 and 3 days after TBI compared to TBI‐Saline control group (*p *<* *.05).

### 3.5 EPO improved cognitive function outcomes after TBI

**Figure 6:**
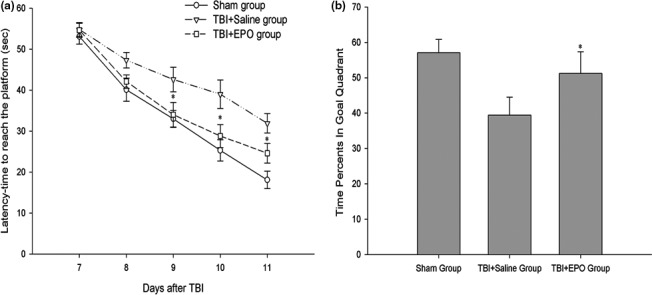
Detect cognitive function by MWM. (a) The spatial learning test at postinjury days 7–11. It shows that latency (time to reach the platform) was significantly shortened during the 5 days test, which suggests that spatial memory was developed in all mice (repeated measures ANOVA,* n *= 10, F = 69.014, *p* < .001). (b) The probe test on postinjury day 12 shows that TBI + EPO group spent significantly higher percentage of time in goal quadrant than the TBI + Saline group. *n *= 10/group, *p < .05 TBI + EPO group versus TBI + Saline group

From 7 to 11 days after TBI, mice were daily evaluated to cognitive function by MWM. The data show that the latency was significantly shortened during the 5 days spatial learning test, suggesting that spatial memory was developed in all mice (F = 69.014, *p* < .05). The mice in TBI + EPO treatment group had significantly reduced latencies to locate the platform compared with the TBI + Saline group at 9–11 days after TBI (*p* < .05, Figure 6a).

In the probe test conducted to assess memory retention at 12 days after TBI, we found that TBI + EPO treatment group spent significantly higher percent of time in the goal quadrant compared to the TBI + Saline group (*p *<* *.05, Figure 6b). The data suggest that EPO treatment significantly improves cognitive function after TBI.

## 4 DISCUSSION

To our knowledge we are the first to report that EPO treatment significantly increased spleen and brain Tregs and anti‐inflammatory factor IL‐10 expression, but decreased proinflammatory factor IL‐1β and TNF‐α expression as well as decreased neutrophils and CD3^+^ T‐cell infiltration into the injured brain tissue. We have also demonstrated that EPO treatment of TBI significantly improves cognitive functional outcome compared to nontreatment TBI controls. The immune/inflammatory modulatory effects may be one of the mechanisms of EPO‐induced neuroprotective effects after TBI.

Cognitive deficit induced by TBI reduces the quality of life in survivors. This deficit may closely relate to the neurodegeneration, which can stem from primary insults as well as the secondary one. Despite the activation of microglia may serve as scavenger, the persistent of this activation can be toxic, which promotes a hostile milieu predisposing to the development of Alzheimer's disease (Ramlackhansingh et al., 2011). The subsequent accumulation of proinflammatory cytokines further aggravates the impairment (Das, Mohapatra, & Mohapatra, 2012). The ability of EPO to improve cognitive outcome has been demonstrated by our study as well as other researchers (Barichello et al., 2014; Yatsiv et al., 2005). Previous studies have shown that EPO promotes angiogenesis and increases cerebral blood perfusion (Meng et al., 2011). However, whether EPO regulates immune/inflammatory effects has not been investigated. In this study, we are the first to demonstrate that EPO treatment reduced brain edema, increased the numbers of Tregs either in periphery or central system, attenuated the infiltration and activation of immune/inflammatory cells, and downregulated the expression of proinflammatory cytokines but upregulated the expression of anti‐inflammatory cytokines.

Brain edema can induce cerebral hernia, decrease cerebral blood flow, and accelerate apoptosis of neurons, which acutely aggravate neurological damage after TBI (Bayir, Kochanek, & Clark, 2003). Our study found that EPO decreased brain edema at 1 and 3 days postinjury, which is consistent with Okutan, Turkoglu, Gok, & Beskonakli (2008) study showing the EPO treatment in cold brain injury decreased brain edema via antiapoptotic and anti‐inflammatory actions. EPO offers strong protection against brain edema, which is partially mediated by diminishing the mitogen‐activated protein kinase pathway (MAPK) activity‐dependent overabundance of aquaporin4 (AQP4) in ischemia and reperfusion‐like injury (Tang et al., 2013).

After primary damages induced by TBI, a large number of immune and inflammatory cells infiltrate into brain tissue through the damaged blood–brain barrier (BBB) (Walsh & Kipnis, 2011). The microglia, inherent immune‐related cell in brain, is also rapidly activated (Cunningham et al., 2014; Glushakova, Johnson, & Hayes, 2014). Both of these partially contribute to brain edema and cognitive deficits after TBI (Xu et al., 2012). In this study, we found that EPO could inhibit MPO^+^ neutrophils infiltration at 1 and 3 days, and suppress CD3^+^ T cells infiltration at 3 and 7 days after TBI. The reason for this disparity may be that neutrophils and CD3^+^ T cells are involved in different stages of the immune/inflammatory reactions (Liesz et al., 2009). Early after TBI, neutrophils not only remove the necrotic cells but also damage some normal cells. Later, CD3^+^ T cells secrete large amounts of proinflammatory cytokines, which exasperate secondary brain injury. In TBI, there is always excessive activation of microglia, which participate in the antigen presentation and promote neuron apoptosis (Wang et al., 2013). In this study, we demonstrated that EPO treatment decreased the activated microglia expression in the cortex of injured brain. EPO also decreased activated microglia expression in autoimmune encephalomyelitis, Alzheimer disease, and ischemic brain injury (Assaraf et al., 2007; Villa et al., 2003; Yuan et al., 2008).

Proinflammatory and anti‐inflammatory cytokines in the brain tissue are mainly produced by T cells, microglia, astrocyte, or other nerve cells after TBI. The balance between proinflammatory and anti‐inflammatory cytokines is associated with prognosis of neurological injury. Excess of proinflammatory cytokines can aggravate the secondary brain injury. Administration of circulating antibody against proinflammatory cytokine TNF‐α protects rat brain from reperfusion injury (Baratz et al., 2011). Anti‐inflammatory cytokine IL‐10 can mediate the neuroprotection of hyperbaric oxygen therapy against TBI (X. Chen et al., 2014). In this study, we found that EPO could reduce proinflammatory cytokines TNF‐α and IL‐1β, but increase anti‐inflammatory cytokines IL‐10 in the brain tissue. The anti‐inflammatory effects may contribute to EPO‐induced neuroprotective effects after TBI.

In addition, we are the first to demonstrate the changes in Tregs in spleen and brain after TBI in mice. Tregs are a subset of T lymphocytes that have the capability to modulate immune/inflammatory function (Schneider, Glenn, & Faunce, 2007). Tregs can reduce brain damage, decrease immune/inflammatory cells infiltration and activation of microglia, downregulates proinflammatory cytokines secretion, as well as upregulate anti‐inflammatory cytokines in ischemia injury (Liesz et al., 2009). We detected that the level of Tregs decreased in periphery and increased in brain tissue at 3 and 7 days after TBI. This could be following the migration of Tregs to injured brain tissue from periphery under attraction of chemotactic factors, such as monocyte chemotactic protein 1 (MCP‐1). Administration of EPO could significantly expand Tregs population both in periphery and central nervous system, which is in agreement with previous report in an animal model of EAE (Yuan et al., 2008). Our data show that EPO treatment not only increases Tregs levels but also decreases neutrophils, CD3^+^ T cells, and microglia, in the injured brain tissue. Therefore, increasing Tregs may play a critical role in neuroprotection and immune/inflammatory modulation induced by EPO treatment in TBI.

Recent clinical trials have demonstrated that the EPO did not significantly improve the neurological recovery in TBI patients (Nichol et al., 2015; Robertson et al., 2014). The discrepancy between these clinical trials and our experiment may be due to the following reasons: the first reason is the different routes and dosages of EPO administration between our experiment and the clinical trials; the second one may be the unavoidable difference between rodents and human beings. They are, after all, two species. Moreover, The C57/BL6 mice that we have being used are from a pure line, whereas the conditions of the TBI patients recruited in the clinical trials are more complicated. Last but not least, our study is focused on the immunomodulatory effects of EPO on brain trauma. Despite the sole employment of EPO may not show significant improvement in patients, the combination of EPO and other immunomodulatory agents may yield different results. Therefore, further broad and in‐depth investigations are indicated.

Our study has some limitations. The dosage of EPO administration should be more detailed and follow a concentration gradient. Our study does not involve the molecular mechanisms of immunomodulatory effects of EPO on TBI, and the discrepancy between the clinical trials and animal experiments (mentioned above), which need our more efforts to investigate and address in the future study.

In summary, the findings of this study add new evidences to the nonhematopoietic neuroprotective effects of EPO. Following TBI, EPO treatment reduces cognitive deficits, brain edema, proinflammatory cytokines, infiltration and activation of immune/inflammatory cells, and increases anti‐inflammatory cytokines. More importantly, EPO elevates Tregs level, which may play a key role in neuroprotection and immune/inflammatory modulation. In the light of increasing evidence of neuroprotection induced by EPO, EPO may serve as an effective treatment for TBI in the future.

## COMPETING INTERESTS

The authors declare that they have no competing interests.
